# Data mining combines bioinformatics discover immunoinfiltration-related gene SERPINE1 as a biomarker for diagnosis and prognosis of stomach adenocarcinoma

**DOI:** 10.1038/s41598-023-28234-7

**Published:** 2023-01-25

**Authors:** Yiyan Zhai, Xinkui Liu, Zhihong Huang, Jingyuan Zhang, Antony Stalin, Yingying Tan, Fanqin Zhang, Meilin Chen, Rui Shi, Jiaqi Huang, Chao Wu, Zhishan Wu, Shan Lu, Leiming You, Jiarui Wu

**Affiliations:** 1 School of Chinese Materia Medica, Beijing University of Chinese Medicine, Beijing, 100029 China; 2 Institute of Fundamental and Frontier Sciences, University of Electronic Science and Technology of China, Chengdu, 611731 China; 3 School of Life Sciences, Beijing University of Chinese Medicine, Beijing, 100029 China

**Keywords:** Bioinformatics, Gene expression analysis

## Abstract

Stomach adenocarcinoma (STAD) is a type of cancer which often at itsadvanced stage apon diagnosis and mortality in clinical practice. Several factors influencethe prognosis of STAD, including the expression and regulation of immune cells in the tumor microenvironment. We here investigated the biomarkers related to the diagnosis and prognosis of gastric cancer, hoping to provide insights for the diagnosis and treatment of gastric cancer in the future. STAD and normal patient RNA sequencing data sets were accessed from the cancer genome atlas (TCGA database). Differential genes were determined and obtained by using the R package DESeq2. The stromal, immune, and ESTIMATE scores are calculated by the ESTIMATE algorithm, followed by the modular genes screening using the R package WGCNA. Subsequently, the intersection between the modular gene and the differential gene was taken and the STRING database was used for PPI network module analysis. The R packages clusterProfiler, enrichplot, and ggplot2 were used for GO and KEGG enrichment analysis. Cox regression analysis was used to screen survival-related genes, and finally, the R package Venn Diagram was used to take the intersection and obtain 7 hub genes. The time-dependent ROC curve and Kaplan–Meier survival curve were used to find the SERPINE1 gene, which plays a critical role in prognosis. Finally, the expression pattern, clinical characteristics, and regulatory mechanism of SERPINE1 were analyzed in STAD. We revealed that the expression of SERPINE1 was significantly increased in the samples from STAD compared with normal samples. Cox regression, time-dependent ROC, and Kaplan–Meier survival analyses demonstrated that SERPINE1 was significantly related to the adverse prognosis of STAD patients. The expression of SERPINE1 increased with the progression of T, N, and M classification of the tumor. In addition, the results of immune infiltration analysis indicated that the immune cells’ expression were higher in high SERPINE1 expression group than that in low SERPINE1 expression group, including CD4^+^ T cells, B cells, CD8^+^ T cells, macrophages, neutrophils and other immune cells. SERPINE1 was closely related to immune cells in the STAD immune microenvironment and had a synergistic effect with the immune checkpoints PD1 and PD-L1. In conclusion, we proved that SERPINE1 is a promising prognostic and diagnostic biomarker for STAD and a potential target for immunotherapy.

## Introduction

Gastric cancer, particularly stomach adenocarcinoma (STAD), is the fifth most common cancer and the third leading cause of cancer death worldwide, with more than 1 million new cases estimated each year. It is also prone to relapse during the late stage and has a high mortality rate^1,2^. As it frequently at advanced stage upon diagnosis in clinical situation, STAD presents a high mortality rate, making it the third leading cause of cancer­related deaths. Risk factors for STAD include but not limited to age, gender, infection of Helicobacter pylori, and high nitrite diet^3,4^. In order to improve diagnostic, prevention, and treatment of STAD, biomarkers or therapeutic targets for the medical conditions need to be explored urgently. In recent years, bioinformatics research related to transcriptomic studies and high-throughput sequencing have led to the discovery of more and more genes involved in driving. A few studies have found a novel prognostic biomarker for STAD by high throughput transcriptome data mining^5^. The tumor microenvironment (TME) refers to the non-cancerous cells and components that are presented in the tumor, including the molecules they produce and release. The constant interactions between tumor cells and the TME play decisive roles in tumor initiation, progression, metastasis, and response to therapies^6^. Abnormalities in the TME may lead to widespread tumor heterogeneity. Therefore, cancer patients’ therapeutic response is influenced by their own TME condition to a large extent^7^. We have identified a predictive biomarker: Serpin family E member 1 (SERPINE1). As a primary inhibitor of uridylyl phosphate adenosine (uPA) and tissue plasminogen activator (tPA), SERPINE is involved in the regulation of these enzymes^8^. Evidences demonstrated that it can promote tumor progression and metastasis as it is promoting the tumor migration, invasion, and angiogenesis^9,10^. To comprehensively understand the role of SERPINE1 in tumor immunity, we used ESTIMATE, CIBERSORT, and ssGSEA (packages) to calculate the proportion of tumor-infiltrating immune cells (TIC), the ratio of immune cells, and expression from the transcriptome profiling data of STAD from The Cancer Genome Atlas (TCGA).

In this study, a comparison of clinical characteristics, immune infiltration, and immune checkpoints was conducted between two groups of patients with the reference of their expression level of SERPINE1. Our results indicated that a high expression of SERPINE1 had stronger immune infiltration than a low expression, suggesting that a high expression of SERPINE1 might respond better to immunotherapy than a low expression of SERPINE1. We have demonstrated SERPINE1 may function as a tumor-associated hub gene in STAD with a potential remarkable impact on the immunological context of STAD.

## Results

### Identification of integrated TCGA DEGs and co-expression network construction

**Figure 1:**
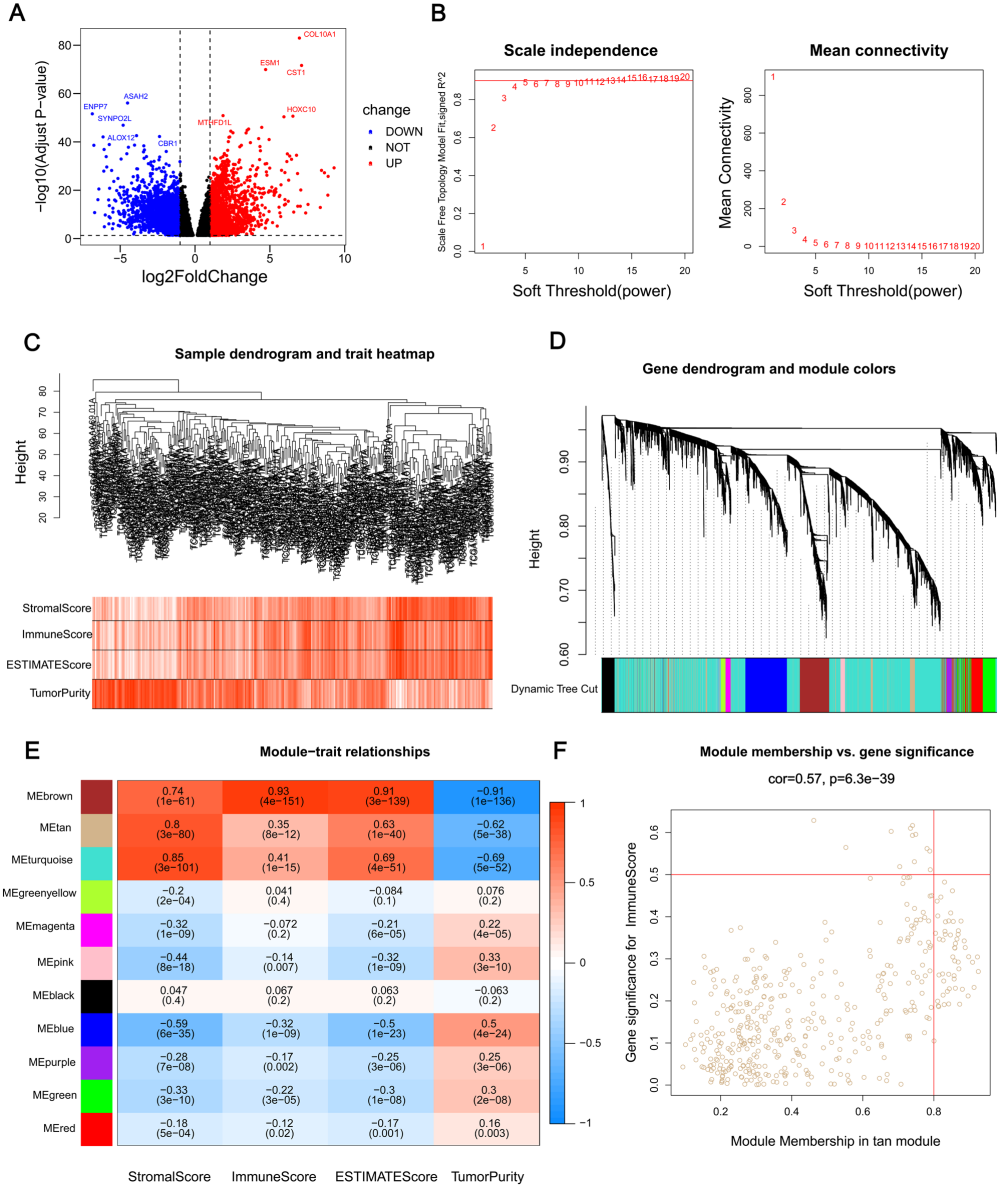
Identification of module genes associated with both clustering and immunity in the WGCNA. (**A**) Volcano plot of differential analysis. (**B**) Analysis of network topology for soft powers. (**C**) Differential genes dendrogram and trait heatmap. (**D**) Dendrogram and genes module colors. (**E**) Heatmap between module eigengenes and ESTIMATE results. (**F**) Scatter plot of module eigengenes in the tan module. The figures were created by R4.1.2 (https://www.r-project.org/).

The differentially expressed genes (DEGs) were accessed by a screening from both STAD samples (n = 375) and normal samples (n = 32). The absolute value of log2 fold change > 2.0 and *p* < 0.01 were defined as rigorous thresholds and 1495 DEGs were screened, which includes 685 upregulated genes and 810 downregulated genes. The volcano plot visually showed the distribution of DEGs in the pre-mentioned STAD samples and normal samples (Fig. 1A). Next, we used the R package WGCNA to calculate the power parameter and cluster of the STAD samples in TCGA. Then, A scale-free topology of the network was then generated by setting the soft-thresholding power to 12 (Fig. 1B). We also drew a cluster tree for the samples (Fig. 1C). We obtained 18 modules in this network, and their relationship was shown in a cluster dendrogram (Fig. 1D). The turquoise module included the maximum of 3268 genes, while the minimum of 73 genes was contained in the green-yellow module. We plotted the heatmap of the module-trait relationships to evaluate the association between each module and the immune score, stromal score, ESTIMATE score and tumor purity (Fig. 1E). Based on the heatmap results, the tan module (436) was selected based onits correlation with the stromal score (R = 0.8, P = 3e^−80^). Gene significance (GS) analysis and module membership (MM) analysis indicated that the genes associated with STAD and the immune were also significantly associated (Fig. 1F).

### Construction of PPI and screening of hub genes

**Figure 2:**
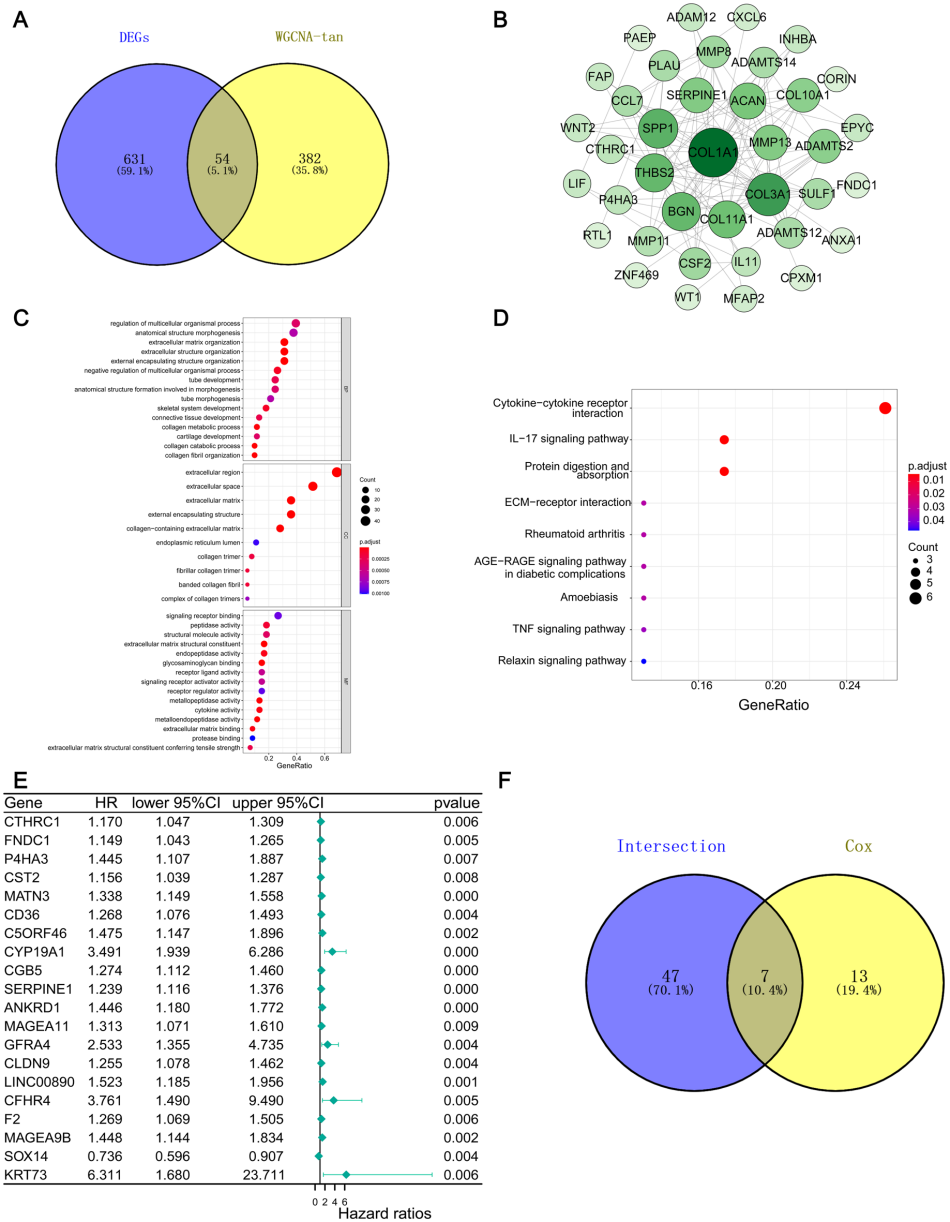
Venn plots, Protein–protein interaction network, enrichment analysis of GO and KEGG for DEGs and Univariate Cox regression analysis. (**A**) Venn plot of the intersection of differential genes and tan modular genes. (**B**) 54 intersection genes interaction network constructed with the nodes with interaction confidence value > 0.4. (**C**) GO enrichment analysis of 54 intersection genes with *p* < 0.05. (**D**) KEGG enrichment analysis of 54 intersection genes with *p* < 0.05. Permission has been obtained from Kanehisa laboratories for using KEGG pathway database^11^. (**E**) 1504 DEGs single Univariate Cox regression analysis, listing the top significant factors with *p* < 0.01. (**F**) The Venn plot of the intersection of the intersection gene was obtained from the A diagram, and the intersection gene was obtained from the E diagram.

To further screen hub genes, intersection analysis was performed between the upregulated DEGs and the tan module. 54 genes were accessed and shown in the Venn plot (Fig. 2A). Based on the STRING database, we constructed a PPI network with Cytoscape software to elucidate the underlying mechanism. The interactions between 379 genes are shown in Fig. 2B. We performed GO and KEGG enrichment analysis to determine the biochemical functions of the 54 genes (Fig. 2C, D). The most significant KEGG pathway was cytokine-cytokine receptor interaction. We conducted a comparation between the key nodes in the PPI network and the top 20 factors identified based on their *p* values in univariate COX regressions, and then implemented the intersection analysis. Seven factors including C5ORF46, CGB5, CST2, CTHRC1, FNDC1, P4HA3, and SERPINE1, overlapped in the above analyses (Fig. 2E, F).

### Prognostic and survival analysis of hub genes

**Figure 3:**
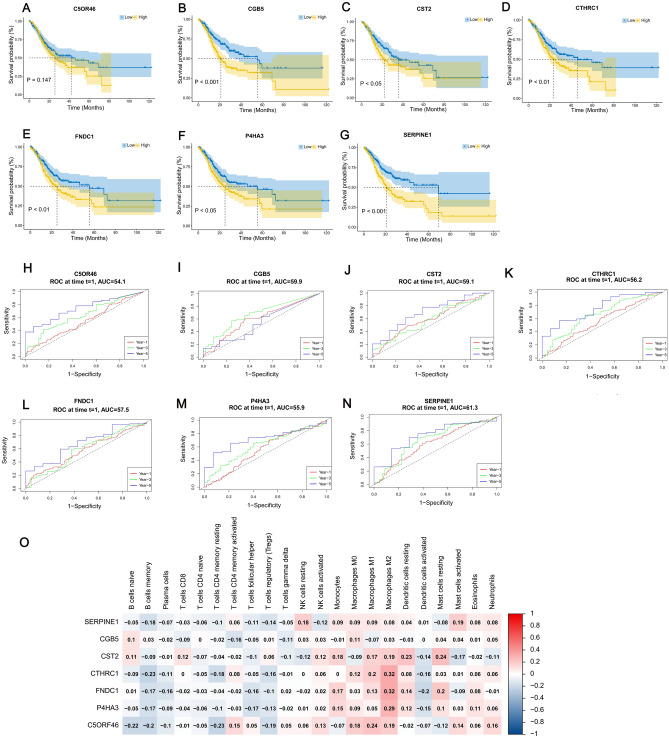
Survival and Prognostic Analysis graphs and heat map for correlation analysis of hub genes. (**A**–**G**) Kaplan–Meier survival curves of 7 hub genes. The horizontal axis indicates the overall survival time in months, and the vertical axis represents the survival rate. *P* value < 0.05 was considered statistically significant. (**H**–**N**) Time-dependent ROC curves of 7 hub genes. Time-dependent ROC curves illustrate the prognostic value of the7 hub genes. (**O**) Heat map of correlation between 7 hub genes and immune cell. The heatmap was created by using “pheatmap” package in R4.1.2 (https://www.r-project.org/).

Prognostic and survival analyses of the seven genes were performed using the R packages timeroc and survival. The packages were used to generate time-dependent ROC curves and Kaplan–Meier curves (Fig. 3A–N). We then examined the correlation between 7 hub genes and immune expression (Fig. 3O). Of the seven genes, Kaplan–Meier analysis showed that higher expression of SERPINE1 was significantly associated with worse overall survival (OS) in STAD patients (*p* < 0.001) (Fig. 3G, N).

### Expression and prognosis of hub genes in pan-cancer

**Figure 4:**
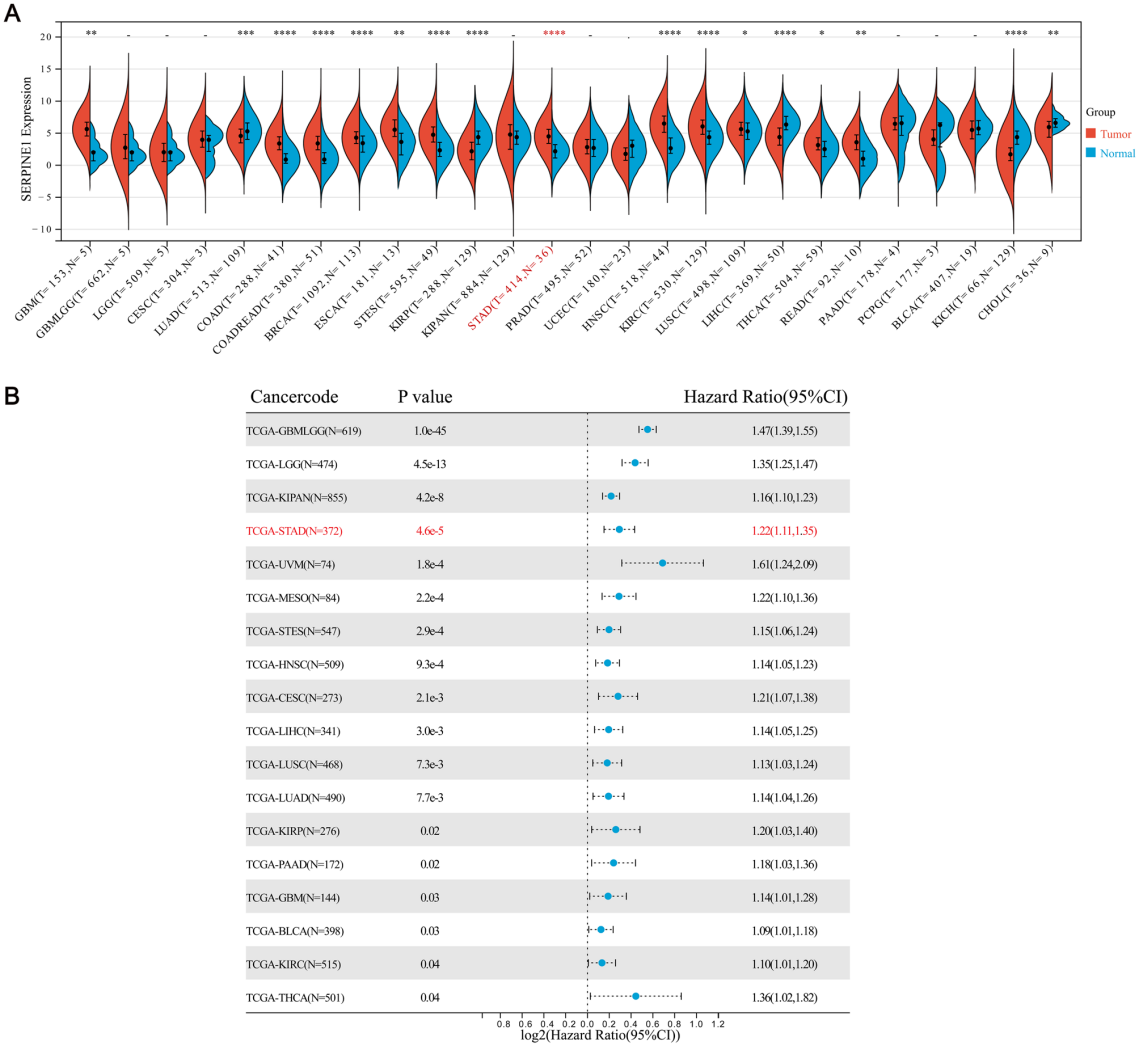
Expression and prognosis of SERPINE1 in pan-cancer. (**A**) Expression of SERPINE1 in pan-cancer. (**B**) Prognosis of SERPINE1 in pan-cancer. The *p* values are labeled using asterisks (*, *p* < 0.05; **, *p* < 0.01; ***, *p* < 0.001; -, not significant, *p* > 0.05).

We first studied the expression of SERPINE1 in the pan-cancer data set of TCGA, and the results showed that the expression levels of SERPINE1 in Colon adenocarcinoma (COAD), Colon adenocarcinoma/ Rectum adenocarcinoma Esophageal carcinoma (COADREAD), Stomach and Esophageal carcinoma (STES), Head and Neck squamous cell carcinoma (HNSC) and STAD were significantly increased (*p* < 0.001), In contrast, SERPINE1 expression in Liver hepatocellular carcinoma (LIHC), Kidney renal papillary cell carcinoma (KIRP), Kidney Chromophobe (KICH) significantly decreased (*p* < 0.001) (Fig. 4A). Then we conducted a single variable cox regression analysis on the relationship between the expression of SERPINE1 and the prognosis of pan-cancer. The results showed that the high expression of SERPINE1 had poor prognosis in Glioma (GBMLGG), Brain Lower Grade Glioma (LGG), Pan-kidney cohort (KIPAN), Uveal Melanoma (UVM), Mesothelioma (MESO), STES, HNSC, Cervical squamous cell carcinoma and endocervical adenocarcinoma (CESC), LIHC, Lung squamous cell carcinoma (LUSC), Lung adenocarcinoma (LUAD) (*p* < 0.01) (Fig. 4B).

### Correlation of SERPINE1 expression with the clinical indicators

**Figure 5:**
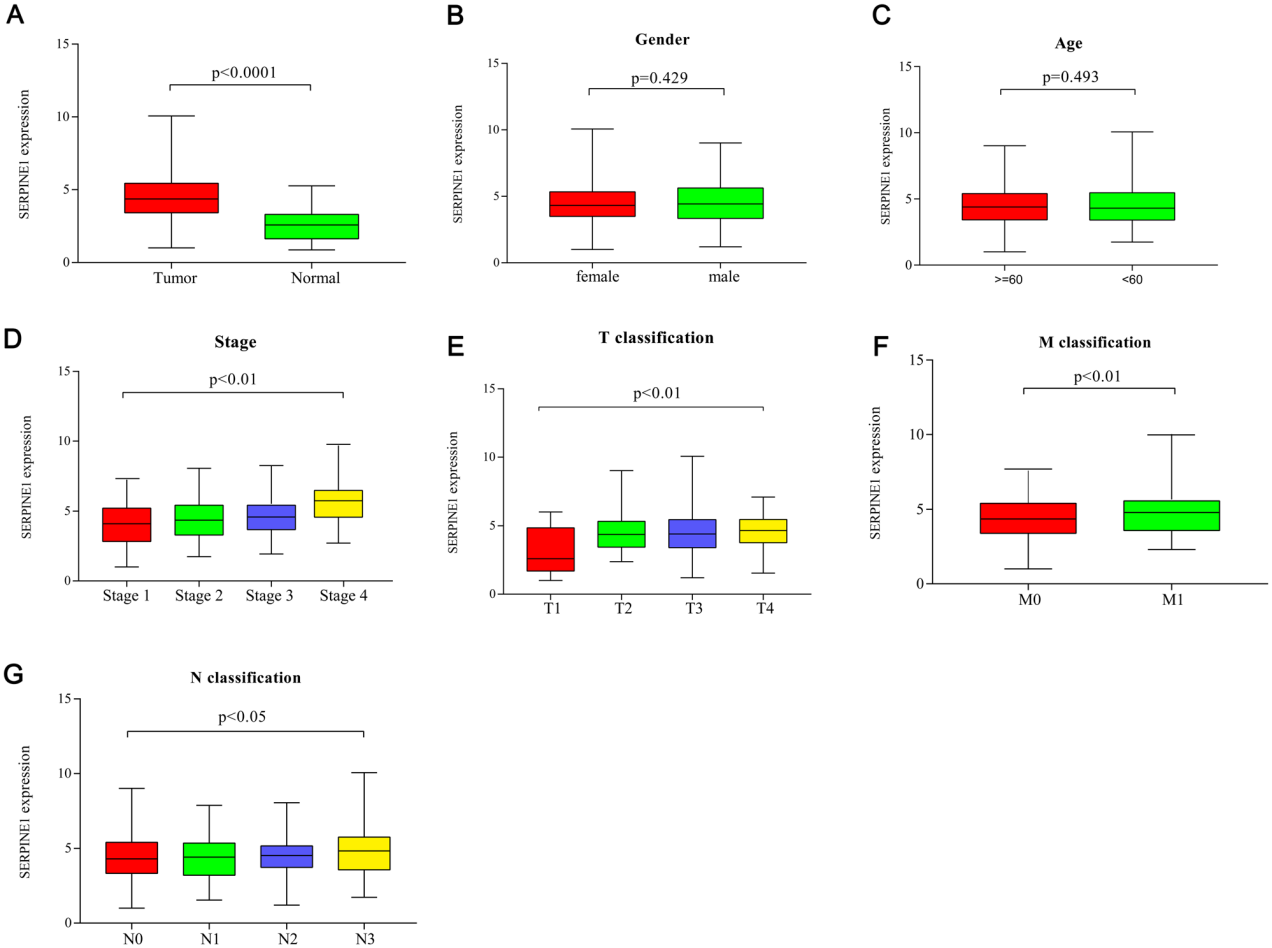
The expression of SERPINE1 in tumor and normal samples and its correlation with clinical indicators. (**A**) Differences in the expression of SERPINE1 in tumor tissues and normal tissues. (**B**) Gender. (**C**) Age. (**D**–**G**) The correlation between the expression of SERPINE1 and clinicopathological staging. Wilcoxon rank sum or Kruskal–Wallis rank sum test served as the statistical significance test.

In the present study, the Wilcoxon rank sum test revealed that the expression of SERPINE1 was significantly higher in tumor samples (*p* < 0.0001) than in normal samples (Fig. 5A). The expressions of SERPINE1 in gender and age of STAD patients did not show significant differences (Fig. 5B, C). However, the expression of SERPINE1 increased with the development of tumor node metastasis classification (*p* < 0.05) (Fig. 5D–G). Results demonstrated that the SERPINE1 expression in TME exhibited a negatively correlation with STAD patients’ prognosis (*p* < 0.05). Notably, the expression of SERPINE1 was significantly different in T and M classifications (*p* < 0.01).

### Correlation of SERPINE1 with immune cell ratio and immune score

**Figure 6:**
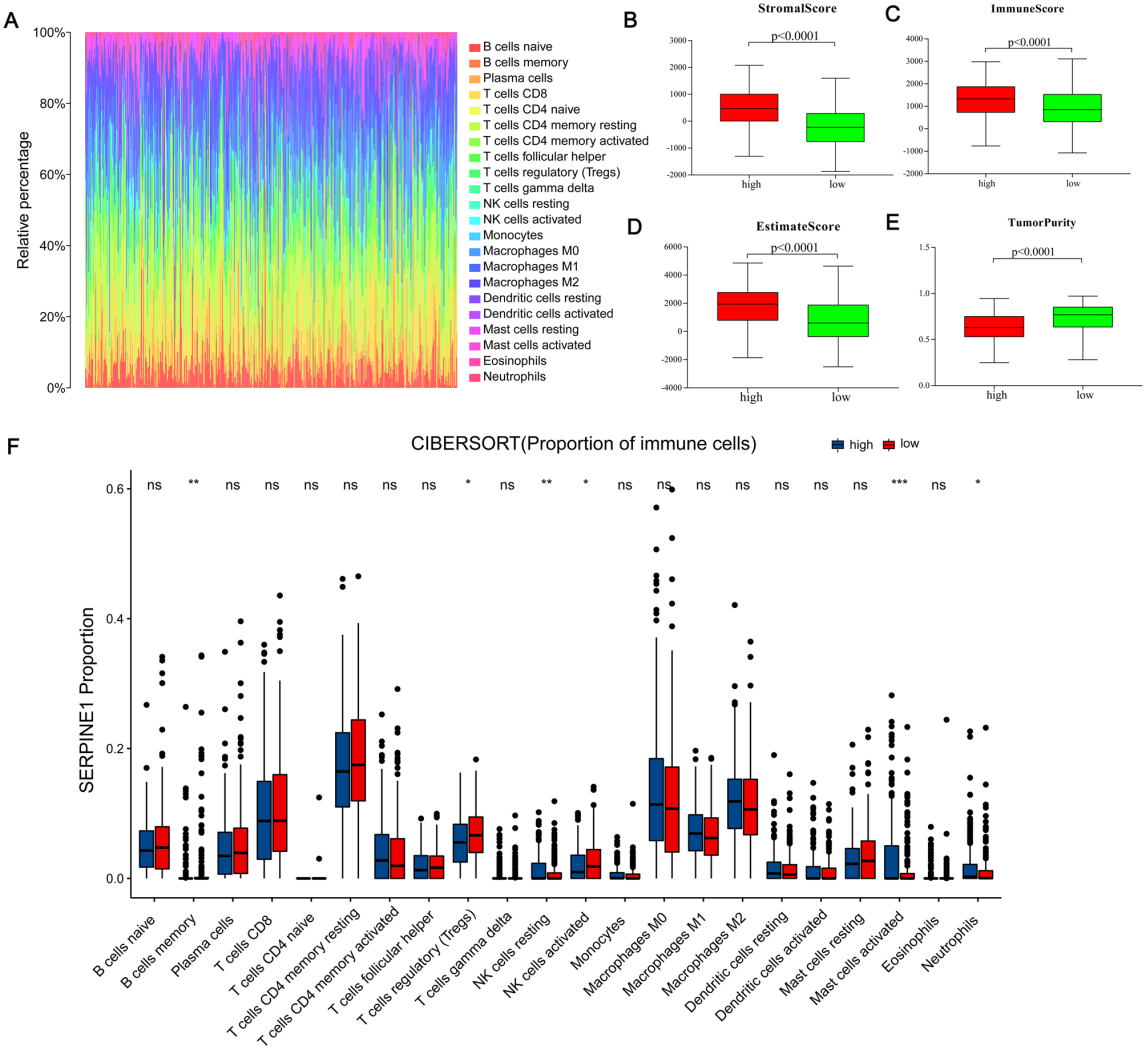
Comparison of Immune Cell Proportion and Immune Score between High and Low Expression of SERPINE1. (**A**) Bar chart of the proportion of 21 immune cells in STAD tumor samples. (**B**) The correlation between the expression of SERPINE1 and the stromal score. (**C**) The correlation between the expression of SERPINE1 and the immune score. (**D**) The correlation between the expression of SERPINE1 and the estimate score. (**E**) The correlation between the expression of SERPINE1 and the tumor purity. (**F**) The correlation between the expression of SERPINE1 and the proportion of immune cells. The P values are labeled using asterisks (^*^p < 0.05, ^**^p < 0.01, ^***^p < 0.001, ns, not significant, *p* > 0.05).

SERPINE1 expression was grouped by level of expression (high vs. low) to confirm the correlation between SERPINE1 expression and immune microenvironment. The T test revealed that the expression of SERPINE1 was significantly higher in the Immune Score, Stromal Score, and ESTIMATE Score groups (*p* < 0.0001) than in the low expression group (Fig. 6B–E). Then, the proportion and expression of immune subsets infiltrating the tumor were analyzed separately using the CIBERSORT and ssGSEA algorithms (Fig. 6A, F). The high-expression group had more B cells, regulatory T cells, and Resting NK cells than the low expression group. It can be concluded, therefore, that the high expression group had a more active immune system than the low expression group.

### Correlation of SERPINE1 with immunoinfiltration

**Figure 7:**
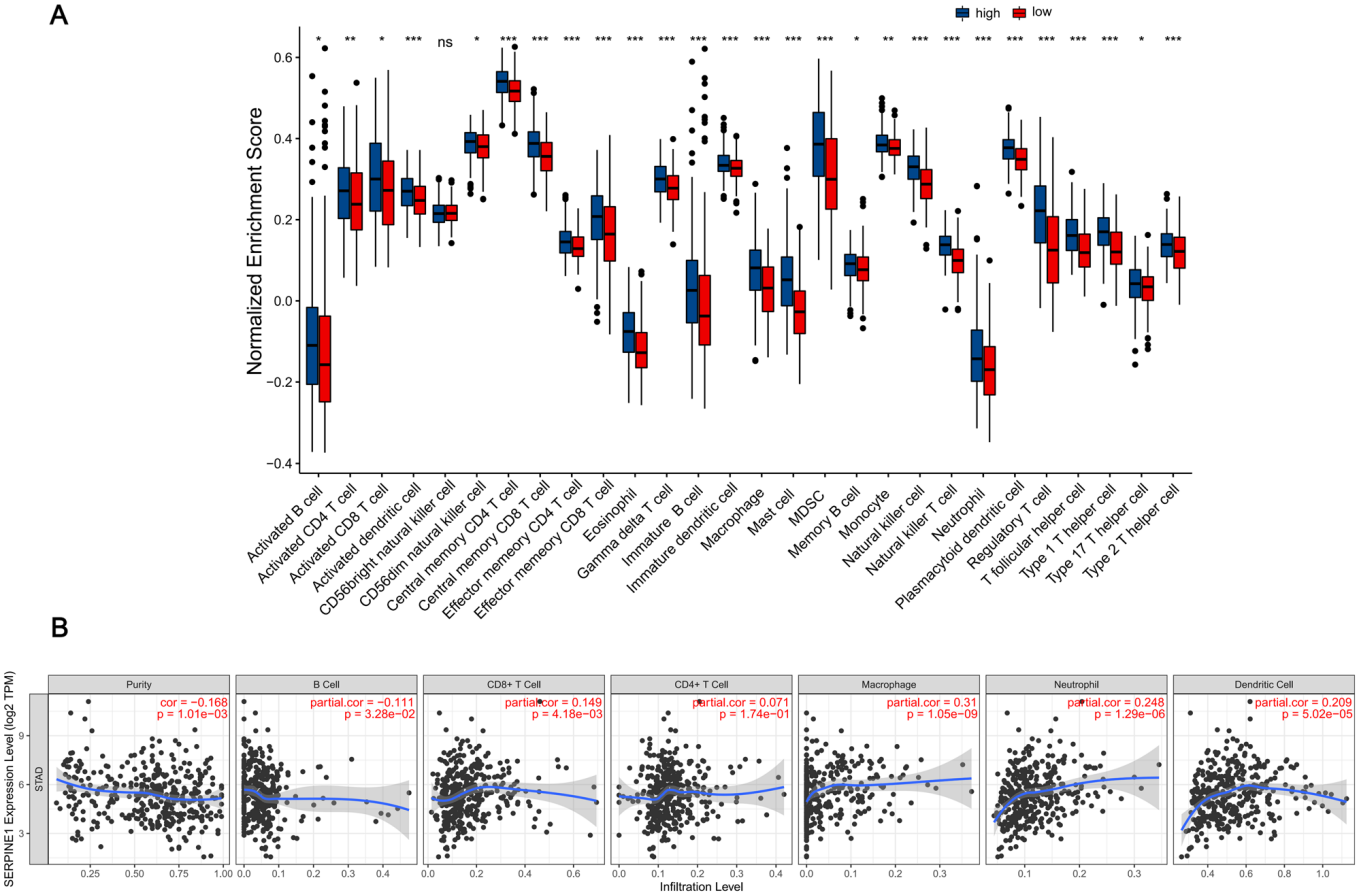
Expression and immunoinfiltration analysis of SERPINE1 in STAD. (**A**) The correlation between the expression of SERPINE1 and the expression of immune cells. (**B**) Correlation of SERPINE1 expression with immune infiltration level in STAD.

In order to more accurately explore the correlation between SERPINE1 expression and immune infiltration, we used the ssGSEA algorithm to analyze the correlation between the expression of SERPINE1 and the expression of 26 immune cells. The results showed that the expression levels of immune cells such as CD4^+^ T cells, B cells, CD8^+^ T cells, macrophages, neutrophils, and others in the SERPINE1 high expression group were higher than those in the SERPINE1 low expression group. (*p* < 0.01) (Fig. 7A). In order to understand the specific effect of SERPINE1 expression on an immune cell, we then used the TIMER database to analyze the correlation between SERPINE1 expression in STAD and tumor purity, B Cells, CD8^+^ T Cells, CD4^+^ T Cells, macrophages, neutrophils, and dendritic cell infiltration levels. The results showed that SERPINE1 expression was significantly negatively correlated with tumor purity and B cell infiltration level (*p* < 0.05). And a significant positive correlation was found between SERPINE1 and infiltration levels of CD8^+^ T cells, macrophages, neutrophils, and dendritic cells in STAD (*p* < 0.01) (Fig. 7B).

### Correlation analysis between SERPINE1 and immune checkpoints

**Figure 8:**
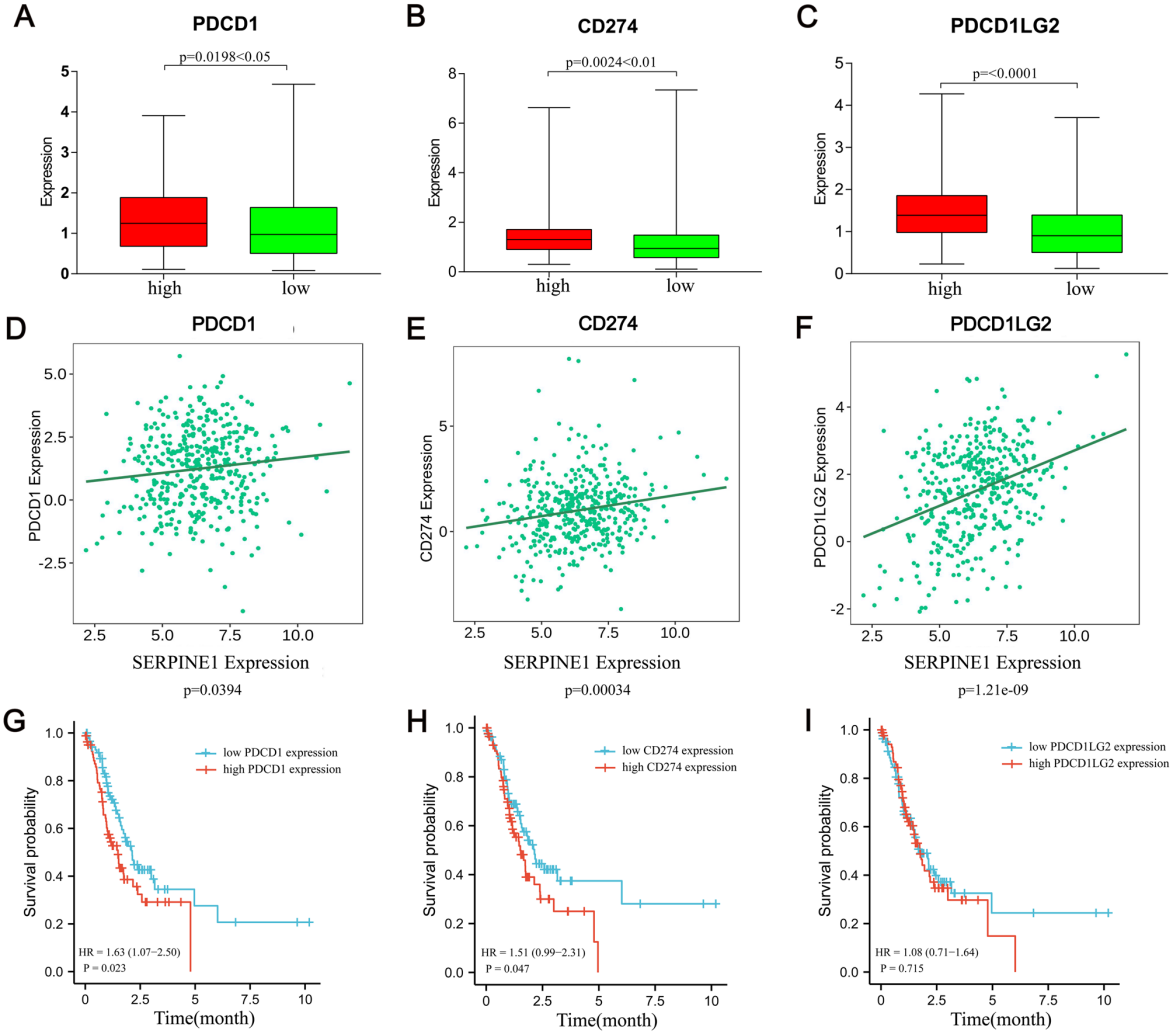
Correlation analysis between SERPINE1 and immune checkpoints. (**A**) The relationship between the high and low expression of SERPINE1 and the expression of PDCD1. (**B**) The relationship between the high and low expression of SERPINE1 and the expression of CD274. (**C**) The relationship between the high and low expression of SERPINE1 and the expression of PDCD1LG2. (**D**) The correlation between the expression of SERPINE1 and the level of PDCD1. (**E**) The correlation between the expression of SERPINE1 and the level of CD274. (**F**) The correlation between the expression of SERPINE1 and the level of PDCD1LG2. (**G**) High expression of SERPINE1 and high and low expression of PDCD1 in STAD patients stratified by Kaplan–Meier survival analysis. (**H**) High expression of SERPINE1 and high and low expression of CD274 in STAD patients stratified by Kaplan–Meier survival analysis. (**I**) High expression of SERPINE1 and high and low expression of PDCD1LG2 in STAD patients stratified by Kaplan–Meier survival analysis. *p* < 0.05 was considered statistically significant.

SERPINE1 may be a candidate immune infiltration for the TME, based on the pre-mentioned analysis results. Considering the importance of immune checkpoints in immunotherapy, A further investigation was conducted into the potential relationship between SERPINE1 and some critical immune checkpoints in the body. Using Spearman correlation analysis, we analyzed the correlation between SERPINE1 and immune checkpoint genes (PD1, CD274, PDCD1LG2) (Fig. 8A–F). In order to understand the relationship between SERPINE1 and immune checkpoint level, we used the TISIDB database to analyze the expression of three immune checkpoint genes and SERPINE1.The results demonstrated that SERPINE1, CD274 and PDCD1LG2 were positively correlated with PD1. In addition, Kaplan–Meier survival analysis showed that patients with high expression of SERPINE1, PDCD1, and CD274 had shorter OS than patients with low expression of SERPINE1, PDCD1, and CD274 (Fig. 8G–I). In the progression of STAD, SERPINE1 may act synergistically with PDCD1, CD274 and other immune checkpoints.

### Functional enrichment analysis of SERPINE1 related genes in STAD

**Figure 9:**
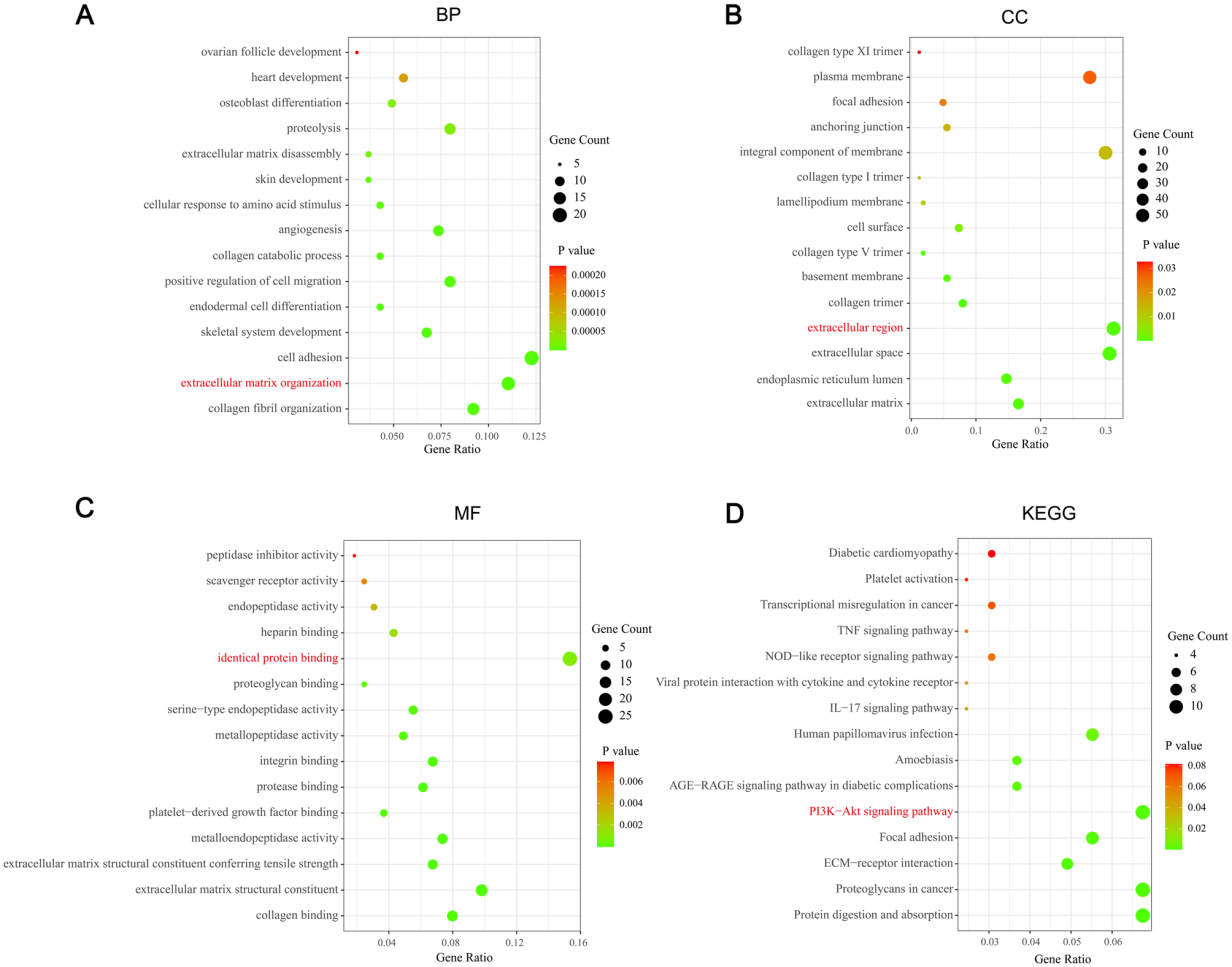
Functional enrichment analysis of SERPINE1 related genes in STAD. (**A**) GO:BP. (**B**) GO:CC. (**C**) GO:MF. (**D**) KEGG. The terms in red were the mainly enriched GO terms and KEGG pathway. Permission has been obtained from Kanehisa laboratories for using KEGG pathway database^11^.

The first 200 genes related to SERPINE1 in STAD were used for GO functional enrichment analysis and KEGG pathway enrichment analysis (Fig. 9). The GO terms of SERPINE1-related genes are mainly enriched in “extracellular matrix organization” (BP, GO: 0030198), “extracellular region” (CC, GO: 0005576), “identical protein binding” (MF, GO: 0042802). The KEGG pathway enrichment analysis of SERPINE1-related genes is mainly concentrated in “PI3K-Akt signaling pathway” (hsa04151).

## Discussion

Gastric adenocarcinoma (gastric cancer) is a common malignancy with a poor prognosis and high mortality. Gastric cancer is caused by cancerous cells in the gut lining that proliferate abnormally and forming ulcers. Since this process is usually slow, it is difficult to detect it at an early stage, so there is always a delay in diagnosis^12^. Unfortunately, despite considerable efforts in clinical and preclinical research, the prognosis of STAD remains disappointing. The nomogram (Supplementary Fig. 1) in this study and related studies showed that the prognosis of patients with advanced gastric cancer is poor, with a 5-year survival rate of only 20–30%^13,14^. In addition to surgery, chemotherapy, radiation therapy, immunotherapy and targeted therapies are also used to treat gastric cancer. They are usually used in combination to treat STAD^15,16^. TME changes are closely related to the development of cancer. Tumor cells can shape their surrounding microenvironment by secreting various cytokines, chemokines, etc., and this process will lead to changes in surrounding cells. Immune cells are an important part of TME and play an important role in related processes. Increasing evidences demonstrated that the interaction between cancer cells and immune cells will eventually promote tumor progression and metastasis^6,17^.

In recent years, immunotherapy for cancer has attracted increasing attention from researchers. In particular, due to the continuous in-depth research of immunological TME^18^. Immunotherapy with checkpoint inhibitors is a novel treatment approach that rapidly entered clinical practice in glioma, but its role in gastric cancer remains poorly defined^19^. In this study, the clinical samples in STAD was screened and the immune genes related to diagnosis and prognosis were accessed from the TCGA database in order to investigate a potential approach to find therapeutic targets for STAD. We used the R package DESeq2 to obtain 1495 DEGs from STAD samples in the TCGA database in the conduction of the key genes screening. Then we used the R package ESTIMATE, to calculate the scores of immune cells and stromal cells, and then the R package WGCNA to obtain the module of interest. We obtained 54 intersection genes of upregulated differential genes and modular genes. These genes are highly expressed in tumor tissues and are also related to immune infiltration. Therefore, we performed GO and KEGG enrichment analysis for these intersection genes. The results of the enrichment analysis showed that these differential genes are closely related to the immune process. We then determined some genes relevant to survival genes through single variable Cox regression analysis. The final key gene SERPINE1 has been obtained from the screening of the intersectional pool between the gene in PPI network and genes that related to survival.

Serine protease inhibitor clade E member 1(SERPINE1) is a member of the Serine protease inhibitor family and a key modulator of the plasminogen/plasminase system^20^. SERPINE1 is a single-chain, non-glycosylated polypeptide chain of 400 amino acids^21^. This gene is a major inhibitor of tissue plasminogen activator (TPA) and urokinase (UPA)^8^. With the development of high throughput sequencing techniques, some related studies have shown that SERPINE1 is abnormally expressed in a variety of cancers. For example, Xiao et al. have reported high SERPINE1 expression is a potential adverse prognostic indicator regarding pancreatic ductal adenocarcinoma or breast cancer^22,23^. Moreover, Yang et al. reported that the high SERPINE1 expression of was also associated STAD cells in itsmigration, invasion, and proliferation^24^. The knockdown experiment showed that knockdown of SERPINE1 can suppress the growth and invasiveness of gliomas in the brain^25^. In this study, the results of Cox regression analysis, Kaplan–Meier survival analysis, and time-dependent ROC curve showed that STAD patients with high expression of SERPINE1 had short overall survival and adverse prognostic effects. In addition, we also explored the expression of SERPINE1 in pan-cancer, and the results showed that SERPINE1 was highly expressed in various cancers. In order to further clarify the relationship between the expression of SERPINE1 and the prognosis of pan-cancer, we conducted a Cox regression analysis, and found that the high expression of SERPINE1 showed adverse prognosis in various cancers. To sum up, we speculate that SERPINE1 is an oncogene and is related to the malignancy degree of STAD. This gene could be a valuable diagnostic and prognostic marker.

Although it has been reported that the behavior and the prognosis of tumor patients may be affected by immune infiltration^26,27^, the interaction mechanism between SERPINE1 and TME is still unclear. Interventions on TME are promising potential treatments for tumors, therefore, we conducted a study on SERPINE1-related immune infiltration in STAD. Because SERPINE1 is an immune-related gene and immune-related genes usually regulate the immune cell behavior. Therefore, The proportions of 22 types of immune cells in STAD’s microenvironment were then assessed using the CIBERSORT algorithm. Subsequently, the SSGSEA algorithm was used to evaluate the expression of 28 immune cells in the microenvironment of STAD. Also, expression levels of CD4^+^ T cells, B cells, CD8^+^ T cells, neutrophils macrophages, and other immune cells were higher in the group with high SERPINE1 expression than in the group with low SERPINE1 expression. CD4^+^ T cells can regulate cytolytic mechanisms or indirectly attack and kill tumor cells through modulation of a number of cytokines, including TME and others^28,29^. In addition, CD4^+^ T cells have been shown to enhance (help) the response of B cells and CD8^+^ T cells^30^. B cells are known to be the major effector cells of humoral immunity. They secrete immunoglobulins and promote T cell responses to inhibit tumor progression^31,32^. Macrophages and neutrophils play an important role in cancer development and metastasis. They can not only fight cancer but also promote cancer, and their mechanism to promote the cancer process is related to the inflammatory response^33,34^. In addition, the results of Timer database analysis showed that SERPINE1 expression was significantly negatively correlated with STAD tumor purity and B cell infiltration level (*p* < 0.05). But it presented a positive correlation with the infiltration levels of CD8^+^ T cells, macrophages, dendritic cells, and neutrophils in STAD (Fig. 8B) (*p* < 0.01). SERPINE1 expression may promote the expression of macrophages, neutrophils and CD8^+^ T Cells in STAD, thereby promoting tumor progression. Therefore, we speculated that SERPINE1 is an adverse prognostic factor in STAD patients and functions as one of the key factors to regulating the immune response. Although the expression of various immune cells is high in TME, their ability is not sufficient to cause an anti-tumor immune response. Therefore, we speculated that high expression of SERPINE1 may lead to the formation of an immune inhibitiory TME, promoting immune escape and tumor growth. In addition, a variety of immune cells may secrete a large number of anti-inflammatory cytokines to inhibit the immunological microenvironment and promote tumor growth^35,36^. In this study, SERPINE1 was shown to be associated with immune cell infiltration in the STAD microenvironment.

Recent studies have shown that immune checkpoints can drive ormal immune homeostasis function to be induced in cancer cells and escape immune cells attack^37–39^. Immune checkpoint inhibitors that block immune checkpoint function have become one of the most effective methods to treat tumors. For example, some patients with advanced melanoma^40–42^, patients with non-small cell lung cancer^43,44^, and patients with renal cell carcinoma^45,46^ have longer overall survival after anti-PD1 therapy compared with traditional methods. Considering the important role of immunotherapy in STAD, we further analysed the correlation between the high expression of SERPINE1 in TCGA database and immune checkpoint genes (PD-1, PD-L1, and PDL2). The results showed that the expression of PDCD1 (PD-1), CD274 (PD-L1) and PDCD1LG2 (PD-L2) were significantly increased in the SERPINE1 high expression group, and the Kaplan–Meier survival curve results showed that the highly expressed PD1PD-L1 had a lower survival probability in the SERPINE1 high expression group. Therefore, we hypothesise that SERPINE1 is a highly expressed and has immune checkpoint genes and possibly synergistic effects with them. The U.S. FDA has also approved two different anti-PD1 drugs (nivolumab and pembrolizumab) for melanoma and non-small cell lung cancer^47^. It cannot be denied that the current research has certain limitations.

The PI3K/AKT signaling pathway is a well-known one that plays an important role in the growth and proliferation of tumor cells. While the PI3K pathway also has various significant functions in the TME, such as the damage of NK cells’ immune monitoring function due to the loss of PI3Kδ^48^. PI3K and AKT can induce the expression of cytotoxic T lymphocyte effector molecules such as perforin, interferon-γ and granzyme^49^. Inhibition of the excessive activation of this signaling pathway may be considered as another potential therapeutic strategy of tumors. In fact, some PI3K inhibitors have already been adopted as treatment in different types of tumors^50^. Interestingly, the enrichment analysis of SERPINE1-related genes shows that most of them are enriched in the PI3K/AKT signaling pathway. This gene is closely related to the TME, the immune checkpoint gene, and the PI3K/ATK signaling pathway. Based on that, a rational hypothesis is that through the PI3K/ATK signaling pathway, this gene may have association with the immune checkpoint-related gene, and thus affect the occurrence and development of STAD.

There are still many deficiencies in our research. First, the clinical conclusions from this study are mainly from the analysis of public databases. Therefore, further clinical research and verification are urgently needed. Secondly, in our study, the sample size from public databases is also limited, which may lead to inaccurate conclusions. Therefore, large samples from multiple data sets are needed to support these conclusions. Third, in the data we obtained from the TCGA database, most of the clinical information about adjuvant treatment before and after the final surgery is unknown. However, they are key factors for prognosis.

## Conclusions

In brief, we screened SERPINE1 from genes related to immunity and survival and further explored its correlation with clinical characteristics and biological functions of STAD patients by bioinformatics methods. The results show that SERPINE1 can be both a promising biomarker for prognosis and diagnosis for STAD and a potential therapeutic target for STAD.

## Methods

### Raw data

Transcriptome RNA-seq data from 407 STAD cases (normal samples, 32 cases; tumor samples, 375 cases) and the corresponding clinical data were accessed from the TCGA public database in GDC data portal (https://gdc.xenahubs.net).

### Identification of differentially expressed genes (DEGs)

Expression profiling data (STADSeq-Counts) were identified using the R package DESeq2, and 1495 DEGs were obtained. Threshold values were set as |log2FoldChange|> 2, and the adjusted P value should be < 0.05. Then, the volcano plot was visualized using the ggplot2 R package^51^.

### Generation of immune-related score

In this study, ESTIMATE is applied as a method realized by the R package estimate^52^ to determine the fractions of stromal and immune cells with the reference of gene expression signatures in tumor samples. We adopted this method in the evaluation of the TME of each STAD patient in association with a stromal score, immune score, and ESTIMATE score. These are indices reflecting stromal content, the extent of immune cell infiltration, and the synthetic mark of stroma and immune, respectively. The purity of the tumor also involved.

### Identification of key co-expression modules using WGCNA

The R package WGCNA^53,54^ was used to screen hub genes and detect coexpression of gene pairs in this study. The weighted values of the correlation coefficients between gene pairs were defined as elements in the gene coexpression matrix. The soft-thresholding function was determined by the power parameter. The dynamic tree cut method was used in the identification co-expressed gene modules. We also adopted a hierarchical clustering approach to construct a dendrogram of the genes with the reference of the dissimilarity of the unsigned topological overlap matrix (TOM). Genes were grouped into different network modules based on the similarity of expression profiles.

Identification of the key co-expression modules by WGCNA co-expression networks followed the approach of network-based gene screening, which can be used to identify potential biomarker candidates and therapeutic targets. The co-expression networks are constructed by adopting the WGCNA R package with the reference of STAD sequencing data.

### Differential expression analysis and interaction with modules of interest

Following the previously mentioned processes, the modules of interest genes were extracted from the coexpression network, and the overlapping genes between the upregulated DEGs and the modules of interest genes were then used to identify potential prognostic and diagnostic genes. The R package Venn diagram was used to present the results^55^.

### Construction of protein–protein intersection (PPI) network

STRING (Search Tool for the Retrieval of Interacting Genes) is an online tool designed for predicting protein–protein interactions (PPI) and constructing a PPI network of specific genes^56^. In this study, genes with a score ≥ 0.4 were selected from the STRING database to build a network model. The result was presented using Cytoscape software (version 3.7.2)^57^.

### Acquisition of SERPINE1 related genes and KEGG enrichment analysis

We used GEPIA (Gene Expression Profiling Interactive Analysis) to obtain SERPINE1-related genes. The results of GO and KEGG enrichment analysis were accessed by R packages clusterProfiler, enrichplot, and ggplot2, and the differences with a *p* value < 0.05 were defined as statistically significant.

### Univariate Cox regression analysis

The R package survival was used to conduct univariate COX regression analysis, and 20 genes were screened with a significant *p* value of < 0.01.

### Screening of hub genes

We intersected the genes obtained from the COX regression with the PPI intersection genes to obtain 7 hub genes.

### Correlation between hub gene expression with the clinical indicators in STAD patients

We used the R language to determine the results. To analyze the correlation between clinical staging and expression of hub genes, we used the Wilcoxon rank sum test and Kruskal–Wallis rank sum test, and significant difference was defined as the *p* value < 0.05.

### Expression and prognosis of SERPINE1 in pan- cancer

We downloaded the unified and standardized pan-cancer data set: TCGA Pan-Cancer (PANCAN, N = 10,535, G = 60,499) from the UCSC Xena database, and further extracted the SERPINE1 gene from it. Expression data in each sample. We used R software to calculate the expression differences between normal samples and tumor samples in each tumor, and used unpaired Student’s t-Test to analyze the differences. We used the R software package survival to analyze the relationship between gene expression and prognosis in each tumor, and used the Logrank test to perform statistical tests to obtain prognostic significance.

### Survival and prognostic analysis

The R packages survival and timeroc were used for survival and prognostic analysis. 375 tumor samples from 315 cases had a detailed survival time record and their survival curve was plotted as a Kaplan–Meier survival curve. The R package timeroc was used to draw time-dependent curves. The *p* value < 0.05 defines as a statistically significant difference.

### Comparison of immunoinfiltration

Based on the grouping of hub gene expression, the R packages ESTIMATE, CIBERSORT and ssGSEA were used to analyze the differences in immune function related to hub genes. Correlation analysis of SERPINE1 expression and tumor purity, B Cells, CD8^+^ T Cells, CD4^+^ T Cells, macrophages, neutrophils, and dendritic cells infiltration levels in STAD was then performed using the Tumor IMmune Estimation Resource (TIMER, https://cistrome.shinyapps.io/timer/).

### Correlation analysis between hub gene and immune checkpoints

We used Spearman correlation to analyze the correlation between hub genes and immune checkpoints.The TISIDB database (cis.hku.hk/TISIDB/) was used to further validate the correlation analysis between SERPINE1 expression and immune checkpoints. In addition, Kaplan–Meier survival analysis was used to analyze the correlation between the group with the high-expression of hub gene and the group with the high-low-expression of immune checkpoints. In this study, a significant difference was defined with a threshold of PSH 0.05.

## Supplementary Information

Supplementary Information.

## Data Availability

This study only conduct analysis based on TCGA public database, with no experiments on humans and/or the use of human tissue samples involved. All data used in this analysis can be found at the Genomic Data Commons (GDC) data portal (https://gdc.xenahubs.net).
