# Supplementary material for: Data mining combines bioinformatics discover immunoinfiltration-related gene SERPINE1 as a biomarker for diagnosis and prognosis of stomach adenocarcinoma
Source: Sci Rep. 2023 Jan 25;13:1373. doi: 10.1038/s41598-023-28234-7 (PMC9876925; doi:10.1038/s41598-023-28234-7)
Supplement: Supplementary file 1 — Supplementary Information. [file 41598_2023_28234_MOESM1_ESM.docx]

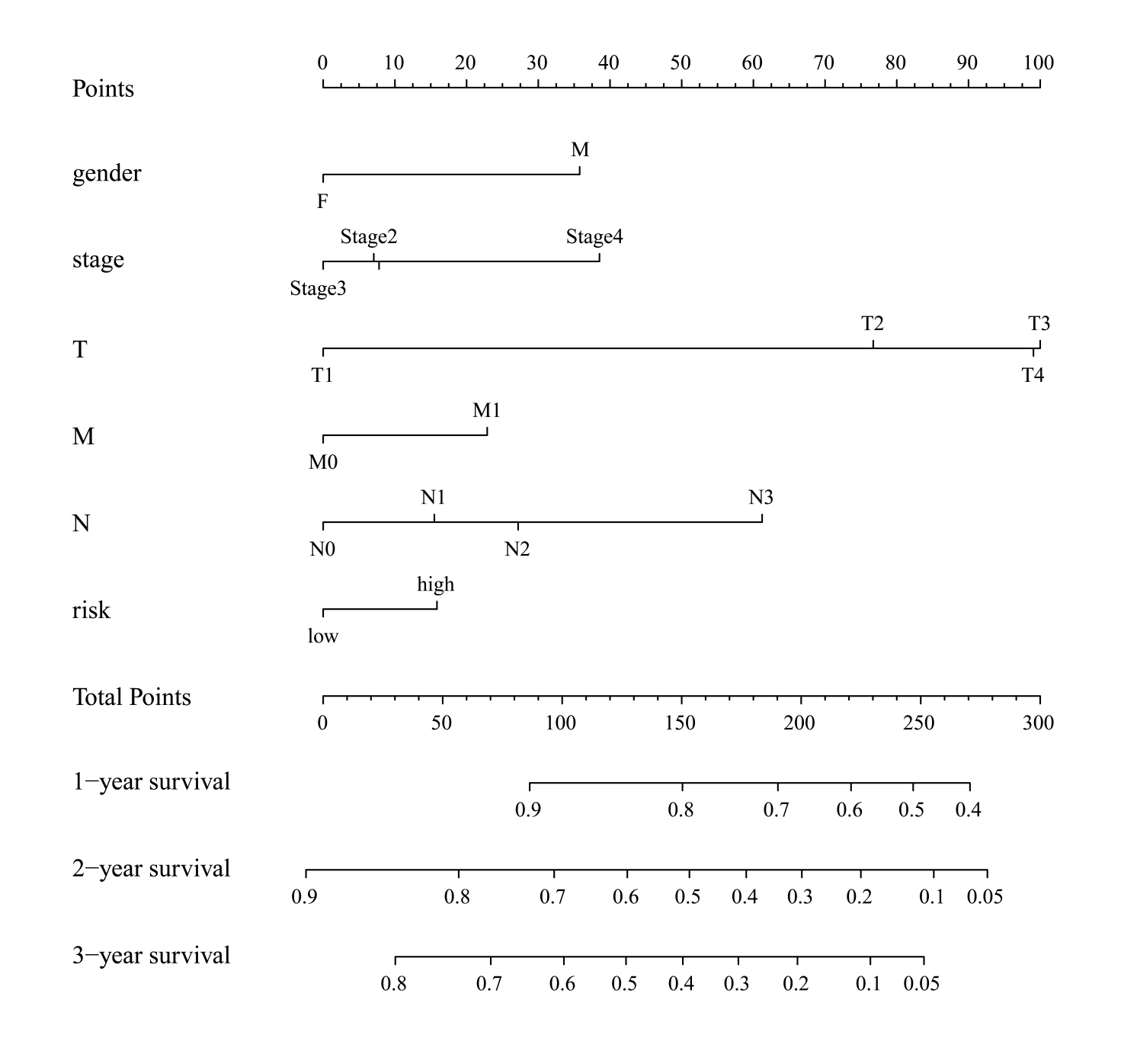


**Supplementary Figure 1** Nomogram Plot. The STAD patient’s clinical indicators correspond to the Points in the first row one by one, and the sum of the scores of each indicator corresponds to the Total Points to predict the STAD patient's 1, 2, and 3-year survival rate.

**
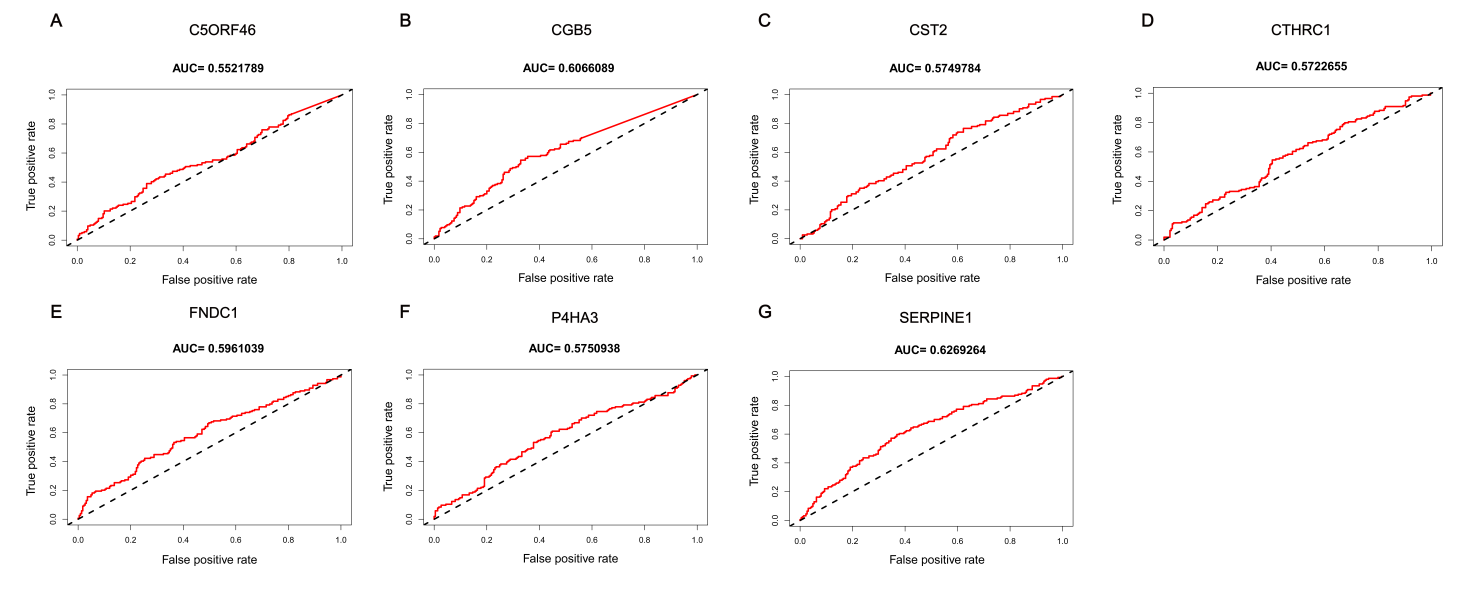
**

**Supplementary Figure 2** Prognostic analysis of 7 hub genes ROC curve. **(A)** C5ORF46. (B)CGB5. **(C)** CST2. **(D)** CTHRC1. **(E)** FNDC1. **(F)** P4HA3. **(G)** SERPINE1.

**
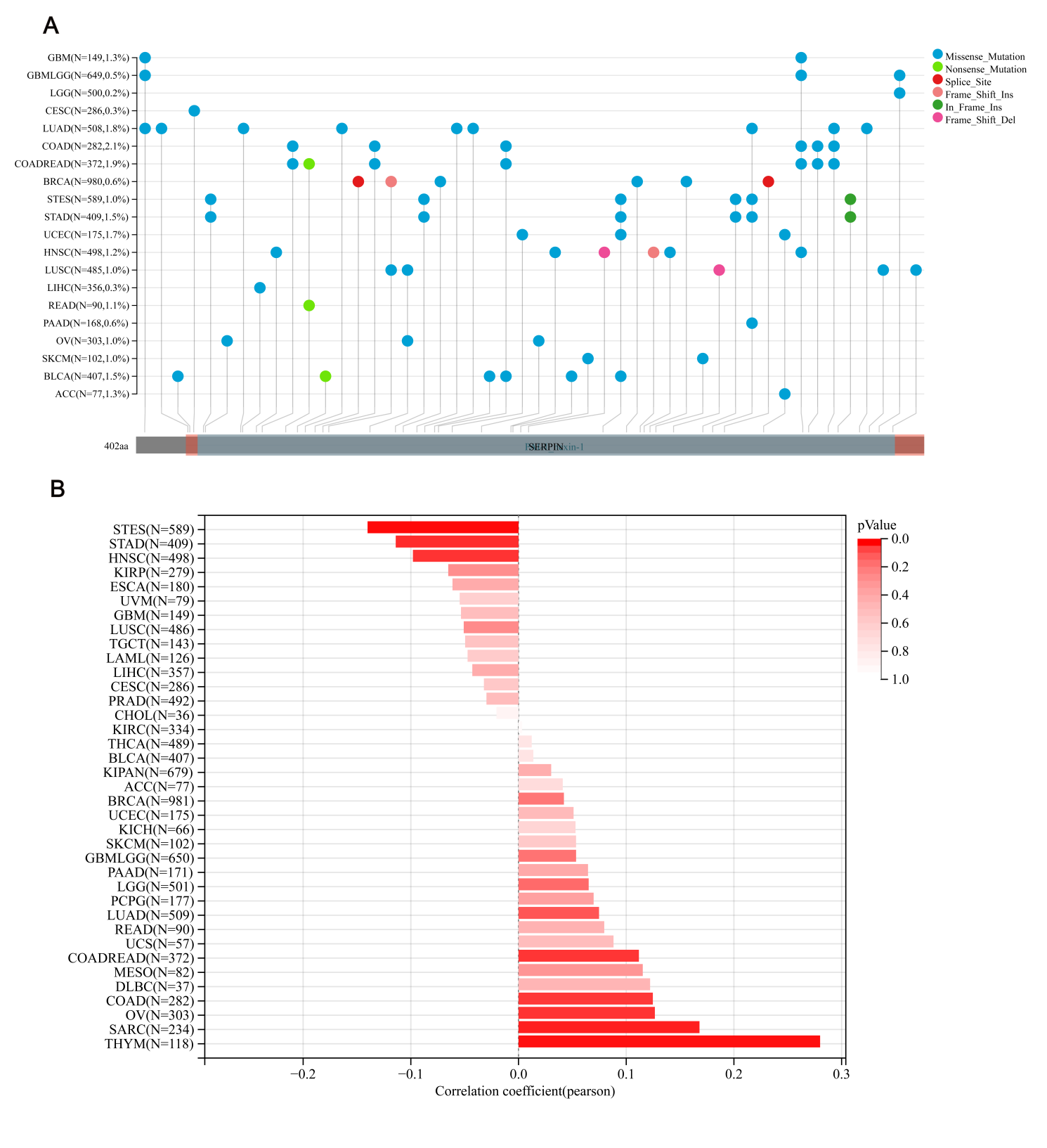
**

**Supplementary Figure 3** Landscape map of gene mutation and correlation map of tumor load mutation of SERPINE1 in pan-cancer. **(A)** Gene mutation landscape map. **(B)** Correlation diagram of tumor load mutation.

**
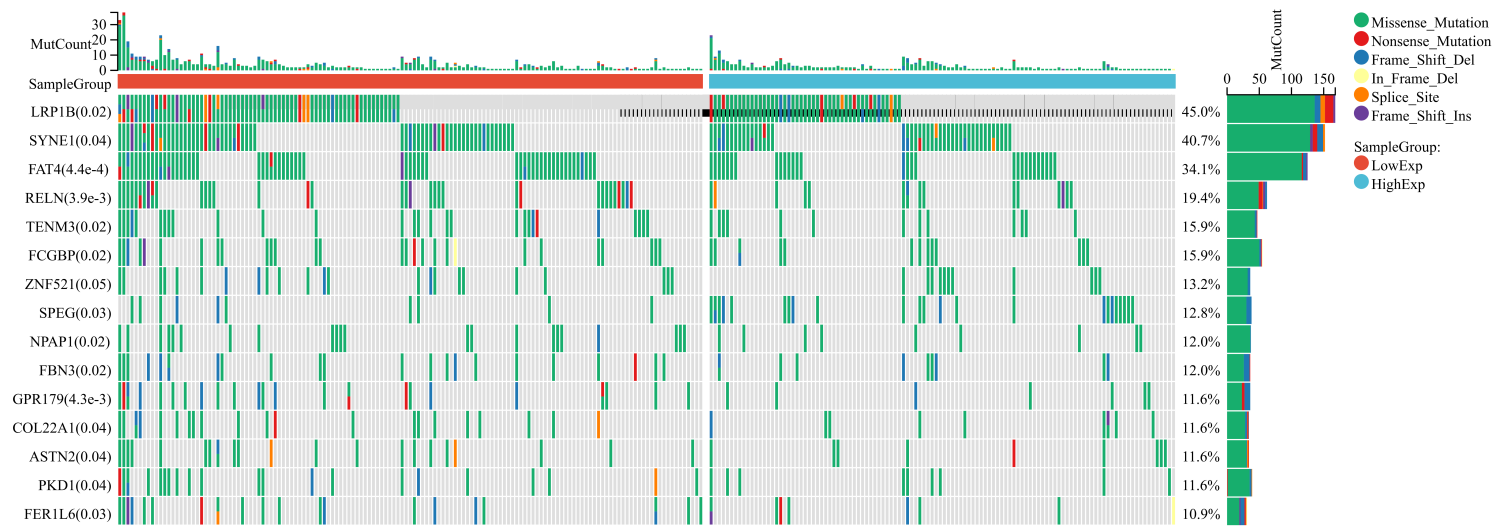
**

**Supplementary Figure 4** Landscape map of the top 15 genes with the highest mutation frequency in STAD
